# Prodromal frontotemporal dementia: clinical features and predictors of progression

**DOI:** 10.1186/s13195-021-00932-2

**Published:** 2021-11-15

**Authors:** Alberto Benussi, Nicholas J. Ashton, Thomas K. Karikari, Antonella Alberici, Claudia Saraceno, Roberta Ghidoni, Luisa Benussi, Henrik Zetterberg, Kaj Blennow, Barbara Borroni

**Affiliations:** 1 Neurology Unit, Department of Clinical and Experimental Sciences, University of Brescia, P.le Spedali Civili 1, 25123 Brescia, Italy; 2 Institute of Neuroscience and Physiology, Sahlgrenska Academy at University of Gothenburg, Mölndal, Sweden; 3 Wallenberg Centre for Molecular and Translational Medicine, University of Gothenburg, Mölndal, Sweden; 4 King’s College London, Institute of Psychiatry, Psychology & Neuroscience, Maurice Wohl Clinical Neuroscience Institute, London, UK; 5 NIHR Biomedical Research Centre for Mental Health & Biomedical Research Unit for Dementia at South London & Maudsley NHS Foundation, London, UK; 6 Molecular Markers Laboratory, IRCCS Istituto Centro San Giovanni di Dio Fatebenefratelli, Brescia, Italy; 7 Clinical Neurochemistry Laboratory, Sahlgrenska University Hospital, Mölndal, Sweden; 8 UK Dementia Research Institute at UCL, London, UK; 9 Department of Neurodegenerative Disease, UCL Institute of Neurology, London, UK

**Keywords:** Frontotemporal dementia, Serum neurofilament light, Prodromal, Mild, Progression, Conversion

## Abstract

**Background:**

The prodromal phase of frontotemporal dementia (FTD) is still not well characterized, and conversion rates to dementia and predictors of progression at 1-year follow-up are currently unknown.

**Methods:**

In this retrospective study, disease severity was assessed using the global CDR plus NACC FTLD. Prodromal FTD was defined to reflect mild cognitive or behavioural impairment with relatively preserved functional independence (global CDR plus NACC = 0.5) as well as mild, moderate and severe dementia (classified as global CDR plus NACC = 1, 2, 3, respectively). Disease progression at 1-year follow-up and serum NfL measurements were acquired in a subgroup of patients.

**Results:**

Of 563 participants, 138 were classified as prodromal FTD, 130 as mild, 175 as moderate and 120 as severe FTD. In the prodromal and mild phases, we observed an early increase in serum NfL levels followed by behavioural disturbances and deficits in executive functions. Negative symptoms, such as apathy, inflexibility and loss of insight, predominated in the prodromal phase. Serum NfL levels were significantly increased in the prodromal phase compared with healthy controls (average difference 14.5, 95% CI 2.9 to 26.1 pg/mL), but lower than in patients with mild FTD (average difference -15.5, 95% CI -28.4 to -2.7 pg/mL). At 1-year follow-up, 51.2% of patients in the prodromal phase had converted to dementia. Serum NfL measurements at baseline were the strongest predictors of disease progression at 1-year follow-up (OR 1.07, 95% CI 1.03 to 1.11, *p* < 0.001).

**Conclusions:**

Prodromal FTD is a mutable stage with high rate of progression to fully symptomatic disease at 1-year follow-up. High serum NfL levels may support prodromal FTD diagnosis and represent a helpful marker to assess disease progression.

**Supplementary Information:**

The online version contains supplementary material available at 10.1186/s13195-021-00932-2.

## Background

Frontotemporal dementia (FTD) encompasses different phenotypes, namely the behavioural variant of FTD (bvFTD) and the agrammatic or the semantic variant of primary progressive aphasia (avPPA and svPPA) [1, 2]. The disease is characterized by a sly onset of executive dysfunctions, behavioural and personality changes, or language impairment [1, 2]. Even though in the last decade the publication of revised clinical criteria and the better definition of FTD phenotypes have substantially improved our understanding of the disease [1, 2], the earliest disease stages and the conversion to fully symptomatic disease, as well as predictors of progression, are still poorly understood.

Prodromal FTD may be defined as the presence of subtle cognitive and/or behavioural changes in the absence of dementia but, unlike the concept of mild cognitive impairment (MCI) in Alzheimer’s disease [3], no detailed characterization has been presented so far [4]. Attempts to describe the earliest phases have recently been made in monogenic FTD, where the study of *at-risk* subjects carrying pathogenetic mutations in *chromosome 9 open reading frame 72* (*C9orf72*), *progranulin* (*GRN*) and *microtubule-associated protein tau* (*MAPT)* has allowed to depict the stages in proximity of dementia conversion [5, 6]. However, in sporadic FTD, the natural history of prodromal disease stages and the rate of conversion to a fully symptomatic disorder have not yet been defined.

Some authors have suggested that a score equal to 0.5 at the global CDR Dementia Staging Instrument plus National Alzheimer’s Coordinating Centre (NACC) behaviour and language domains (CDR plus NACC FTLD—formerly known as FTLD-CDR), may be useful to define prodromal FTD [7–9]. Moreover, a few studies in subjects with genetic prodromal FTD have shown that concentrations of neurofilament light (NfL), a marker of neurodegeneration [10–14] and FTD severity [12, 15–17], may be related to disease progression and conversion to dementia [18, 19]. The possible role of NfL in prodromal sporadic FTD as a prognostic marker of conversion to dementia has not yet been explored, thus preventing the design of evidence-based interventional strategies on disease progression.

These observations prompted the present retrospective study, carried out in a large cohort of FTD patients sub-grouped according to the global CDR plus NACC FTLD scale, which was aimed at (1) characterizing the clinical and behavioural features of prodromal FTD, as compared to more advance stages of FTD; (2) defining the rate of conversion of prodromal FTD to fully symptomatic disease at one-year follow-up; and (3) assessing the predictors of progression, considering serum NfL levels.

## Methods

### Participants

This retrospective study included a consecutive sample of 563 participants from the Centre for Neurodegenerative Disorders, Department of Clinical and Experimental Sciences, University of Brescia, Italy.

Each participant underwent a neurological evaluation, routine laboratory examination and a standardized neuropsychological and behavioural assessment, as previously reported [20].

In all FTD cases, the diagnosis was supported by brain structural imaging, while CSF concentrations of tau, p-Tau_181_ and Aβ_1-42_ or PET amyloid were measured in a subset of cases (*n* = 223), to rule out Alzheimer’s disease, as previously reported [21]. Furthermore, in familial cases (based on the presence of at least one dementia case among first-degree relatives) and early onset sporadic cases, genetic screening for *GRN*, *C9orf72* and *MAPT* P301L mutations was performed; given the low frequency of *MAPT* mutations in Italy [22], we considered only the P301L mutation and we sequenced the entire *MAPT* gene only in selected cases.

### Clinical evaluation

At baseline, patients underwent a standardized neuropsychological battery which included the Mini-Mental State Examination (MMSE), the short story recall test, the Rey complex figure (copy and recall), phonemic and semantic fluencies, the token test, the clock-drawing test and the trail making test (part A and part B) [23].

The level of functional independence was assessed with the basic activities of daily living (BADL) and the instrumental activities of daily living (IADL) questionnaires, whereas neuropsychiatric and behavioural disturbances were evaluated with the Frontal Behaviour Inventory (FBI) [24].

Disease severity was assessed using the global CDR plus NACC FTLD [8, 9]. Prodromal FTD was defined to reflect mild cognitive or behavioural impairment with relatively preserved functional independence, corresponding to a global CDR plus NACC = 0.5; patients with a mild dementia syndrome were classified with a global CDR plus NACC = 1; patients with a moderate or severe dementia syndrome were classified with a global CDR plus NACC = 2 or 3, respectively [8]. To confirm the diagnosis of prodromal FTD, all patients had a follow-up evaluation that confirmed eventual conversion to dementia, or were carriers of a pathogenic FTD mutation.

Disease progression was defined as a transition to a higher global CDR plus NACC score at 1-year follow-up, whereas no progression was defined when an equal or reduced global CDR plus NACC score was recorded at follow-up. One-year follow-up data was available for 258 participants.

### NfL measurements

A subgroup of patients (*n* = 192) underwent blood collection for serum NfL measurement; 63 healthy controls (HC) (age 67.0, IQR 61.0–74.0 years) were recruited among spouses or caregivers as reference.

Serum was collected by venipuncture, processed and stored in aliquots at – 80 °C according to standardized procedures. Serum NfL was measured by single-molecule array (Simoa) technology on an HD-X Analyzer using the commercial NF-light® assay according to the manufacturer’s instructions (Quanterix, Billerica, MA). Detailed analytical procedures and assay validation have been previously described [15]. The lower limits of quantitation for serum NfL were 0.174 pg/mL. The measurements were performed out in one round of experiments using the same batch of reagents, and the operators were blinded to all clinical information. Quality control samples had intra-assay and inter-assay coefficients of variation of less than 8 and 20%, respectively.

### Statistical analysis

Continuous and categorical variables are reported as median (interquartile range) and *n* (%) respectively. Baseline demographic and clinical variables were compared across groups using one-way Kruskal-Wallis or Fisher’s tests, as appropriate. Differences in NfL levels between groups were compared by using the rank-based nonparametric analysis of covariance (ANCOVA) method, with age as a covariate [25]. We reported marginal mean differences with 95% confidence intervals (95% CI) for relevant comparisons.

To assess the contributions of patient characteristics (sex, age, education, presence of genetic mutation, disease duration, clinical phenotype, FBI, MMSE) and NfL levels to disease progression, we developed multilevel univariable and multivariable logistic regression models considering the time- and severity-dependent nature of independent variables throughout different global CDR plus NAAC FTLD groups. To avoid overfitting in the model, variables were chosen based on previous findings [19, 26–30]. Moreover, multicollinearity was checked and only variables with a variance inflation factors (VIF) < 3 were included. Linearity of the continuous variables with respect to the logit of the dependent variable was assessed via the Box-Tidwell procedure [31]. Based on this assessment, all continuous independent variables were found to be linearly related to the logit of the dependent variable. For each factor, odds ratios (ORs) and 95% confidence intervals (CIs) are reported.

**Fig. 1:**
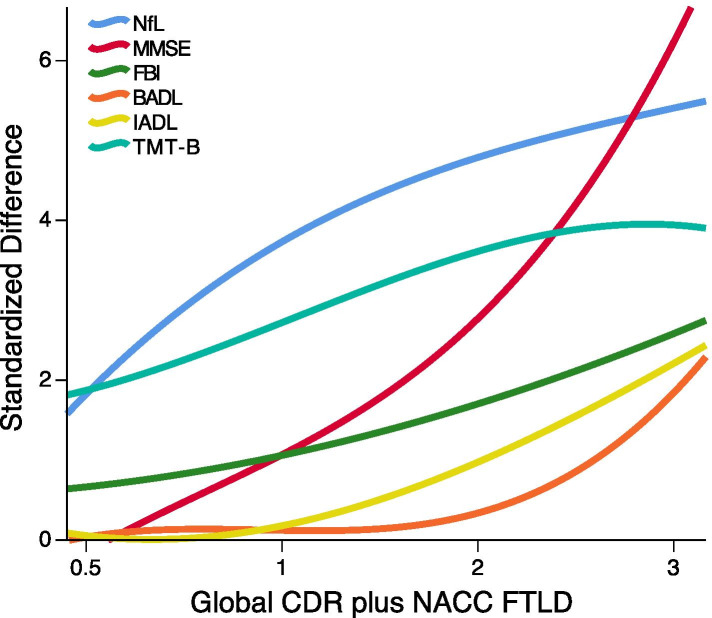
Evolution of severity scores according to the global CDR plus NACC FTLD. Symptom severity in FTLD patients grouped according to the global CDR plus NACC FTLD. NfL = neurofilament light chain; CDR plus NACC FTLD = clinical dementia rating plus National Alzheimer’s Coordinating Center behaviour and language domains FTLD; MMSE = mini mental state examination; FBI = frontal behavioural inventory; BADL = basic activities of daily living; IADL = instrumental activities of daily living; TMT-B = trial making test part B

For Fig. 1, we calculated standardized differences compared with HC (for NfL measures) or published Italian normative data for neuropsychological tests [32, 33].

A two-sided *p* value < 0.05 was considered significant and corrected for multiple comparisons using the Benjamini-Hochberg false discovery rate (FDR) [34]. Data analyses were carried out using IBM SPSS 25.0 and GraphPad Prism 8.0 software.

## Results

### Participant characteristics

In total, 563 participants (median [IQR] age 66.4 [60.5–72.4] years; 296 males [52.6%]) were included in the present study. Of these, 138 were classified as prodromal FTD (global CDR plus NACC FTLD = 0.5), 130 as mild FTD (global CDR plus NACC FTLD = 1), 175 as moderate FTD (global CDR plus NACC FTLD = 2) and 120 as severe FTD (global CDR plus NACC FTLD = 3). Demographic and neuropsychological characteristics of included patients for each subgroup are reported in Table 1.

**Table 1:** Demographic and clinical characteristics of FTLD patients grouped according to the global CDR plus NACC FTLD

Variable	All	Global CDR plus NACC FTLD				
		0.5	1	2	3	***p*** value
Number	563	138	130	175	120	
Age, years	66.4 (60.5–72.4)	65.9 (59.0–71.5)	68.6 (62.3–74.2)	65.4 (59.8–71.2)	66.7 (61.4–73.8)	0.022
Sex male, *n* (%)	296 (52.6)	80 (58.0)	72 (55.4)	91 (52.0)	53 (44.2)	0.143
Education, years	8.0 (5.0–13.0)	8.0 (5.0–13.0)	8.0 (5.0–13.0)	8.0 (5.0–12.0)	8.0 (5.0–12.8)	0.195
Age at onset, years	63.0 (58.0–69.0)	63.0 (56.0–69.0)	65.0 (60.0–71.3)^c^	62.0 (57.0–68.0)^b^	63.5 (57.3–70.0)	0.017
Disease duration, years	2.3 (1.5–3.7)	1.9 (1.2–3.1)^cd^	2.0 (1.3–3.1)^cd^	2.6 (1.6–4.0)^ab^	3.2 (1.9–4.9)^ab^	< 0.001
Monogenic disease, *n* (%)	109 (19.4)	26 (18.8)	23 (17.7)	34 (19.4)	26 (21.7)	0.884
Serum NfL (pg/mL)*	42.6 (30.5–54.9)	26.9 (20.0–39.4)^bcd^	40.8 (28.5–54.8)^a^	44.1 (36.9–60.7)^a^	52.7 (44.0–66.5)^ab^	< 0.001
bvFTD/avPPA/svPPA, *n*	392/108/63	92/25/21	89/27/14	121/35/19	90/108/63	0.602
CDR plus NACC FTLD SB	5.5 (3.0–10.0)	2.5 (1.0–3.5)^bcd^	4.0 (3.0–5.0)^acd^	7.5 (6.0–9.5)^abd^	13.0 (11.0–15.0)^abc^	< 0.001
MMSE	22.2 (16.3–25.4)	28.0 (26.0–29.0)^bcd^	25.0 (22.0–27.3)^acd^	21.0 (17.0–25.0)^abd^	12.5 (4.3–19.0)^abc^	< 0.001
FBI	15.0 (8.0–26.0)	8.0 (3.0–11.0)^bcd^	12.0 (7.0–18.0)^acd^	20.0 (12.0–26.0)^abd^	31.0 (24.3–36.8)^abc^	< 0.001
BADL lost	0.0 (0.0–1.0)	0.0 (0.0–0.0)^cd^	0.0 (0.0–0.0)^d^	0.0 (0.0–1.0)^ad^	3.0 (1.0–5.0)^abc^	< 0.001
IADL lost	1.0 (0.0–3.0)	0.0 (0.0–0.0)^cd^	0.0 (0.0–1.0)^cd^	2.0 (1.0–3.0)^abd^	5.0 (4.0–7.0)^abc^	< 0.001
Short story	7.2 (4.9–10.0)	9.5 (7.7–13.0)^bcd^	8.4 (6.0–11.0)^acd^	6.0 (3.5–8.0)^ab^	5.4 (3.9–7.6)^ab^	< 0.001
Rey figure copy	24.0 (14.8–30.5	30.1 (26.0–32.6)^cd^	27.8 (21.9–32.6)^cd^	21.0 (14.5–29.0)^abd^	12.6 (6.9–18.6)^abc^	< 0.001
Rey figure recall	8.0 (4.5–24.0)	11.8 (8.8–16.5)^bcd^	9.8 (6.5–13.3)^acd^	6.3 (3.5–9.5)^ab^	5.3 (3.2–7.3)^ab^	< 0.001
Phonological fluency	17.0 (10.0–24.0)	23.6 (18.4–29.0)^bcd^	19.5 (14.0–29.0)^acd^	14.9 (9.0–21.0)^abd^	9.4 (4.9–15.1)^abc^	< 0.001
Semantic fluency	22.0 (13.5–31.0)	31.1 (24.0–37.0)^bcd^	27.0 (20.0–33.0)^acd^	19.0 (13.0–25.0)^abd^	10.6 (4.3–18.0)^abc^	<0.001
Digit span	4.5 (3.8–5.5)	5.0 (4.5–5.8)^cd^	4.8 (4.3–5.5)^d^	4.5 (3.8–5.3)^ad^	3.6 (2.8–4.5)^abc^	< 0.001
Token test	26.7 (21.2–29.8)	30.0 (27.9–32.3)^bcd^	28.3 (26.0–30.2)^acd^	24.0 (19.5–27.8)^abd^	18.3 (12.8–24.4)^abc^	< 0.001
Trail making test part A	92.0 (47.0–197.0)	50.0 (28.0–73.0)^bcd^	61.5 (40.8–109.3)^acd^	107.0 (66.0–272.0)^abd^	249.5 (133.3–391.0)^abc^	< 0.001
Trail making test part B	383.0 (198.0–420.0)	191.0 (104.0–358.8)^bcd^	340.0 (130.8–412.0)^acd^	403.0 (337.0–432.0)^ab^	403.0 (377.3–430.8)^ab^	< 0.001
Clock drawing	5.0 (3.0–8.0)	8.0 (6.0–9.0)^bcd^	7.0 (5.0–9.0)^acd^	5.0 (3.0–6.0)^abd^	3.0 (0.0–4.0)^abc^	< 0.001

*NfL* Neurofilament light chain, *CDR Plus NACC FTLD SB* Clinical dementia rating plus National Alzheimer’s Coordinating Center behaviour and language domains FTLD sum-of-boxes, *MMSE* Mini mental state examination, *FBI* Frontal Behavioural Inventory, *BADL* Basic activities of daily living, *IADL* Instrumental activities of daily living. Results are expressed as median (interquartile range), unless otherwise specified

*Results for serum NfL are reported for a subgroup of patients; ^a^significant difference *vs* CDR plus NACC FTLD of 0.5; ^b^significant difference *vs* CDR plus NACC FTLD of 1; ^c^significant difference *vs* CDR plus NACC FTLD of 2; ^d^significant difference *vs* CDR plus NACC FTLD of 3

Genetic and sporadic cases in each subgroup showed comparable demographic and clinical features, except for age at disease onset and age at evaluation (see Supplementary Table 1).

Regarding different phenotypes, we included 392 bvFTD, 108 avPPA and 63 svPPA patients. We observed a similar distribution of phenotypes between global CDR plus NACC FTLD subgroups (see Table 1).

### Prodromal FTD features and FTD evolution

The evolution of functional impairment and neuropsychological symptoms across severity groups are presented in Fig. 1. In the prodromal and mild phases, we observed an early and rapid increase in serum NfL levels, which tended to plateau in the moderate/severe phases. FBI scores were already mildly altered in the prodromal phase and continued to steadily increase in the moderate/severe phases. Considering the extensive neuropsychological assessment, the earliest neuropsychological test to become impaired was the trail making test part B, which was already altered in the prodromal phase, plateauing in the moderate/severe disease phase. On the contrary, MMSE scores steadily deteriorated throughout the whole severity spectrum, particularly in moderated/severe phases. The mild and moderate phases of FTD were characterized by preserved autonomy in BADL and IADL, which were impaired only in severe stages (see Fig. 1).

**Fig. 2:**
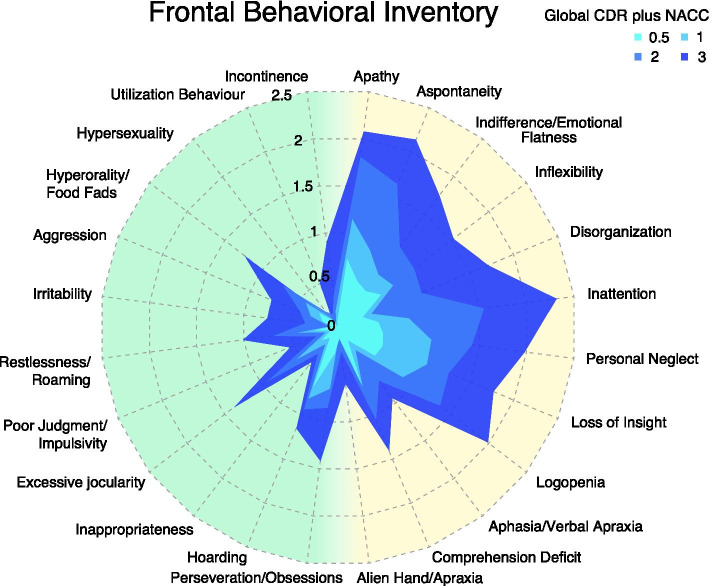
Radar plot of FBI subscores according to the global CDR plus NACC FTLD. Positive symptoms (FBI part A) are reported on the right (light yellow), negative symptoms (FBI part B) are reported on the left (light green). CDR plus NACC FTLD = clinical dementia rating plus National Alzheimer’s Coordinating Center behaviour and language domains; FBI = frontal behavioural inventory

Single items of the FBI for each disease severity group are reported in Fig. 2. In the prodromal phase, the most prominent symptoms were mainly negative behaviours (FBI part A) as apathy, aspontaneity, indifference, inflexibility, inattention, personal neglect, loss of insight, logopenia and comprehension deficit. Regarding disinhibitory symptoms (FBI part B), hoarding was the most relevant. Negative behaviours were the most prominent symptoms also in the mild, moderate and severe stages (see Fig. 2).

### Serum NfL in global CDR plus NACC FTLD subgroups

**Fig. 3:**
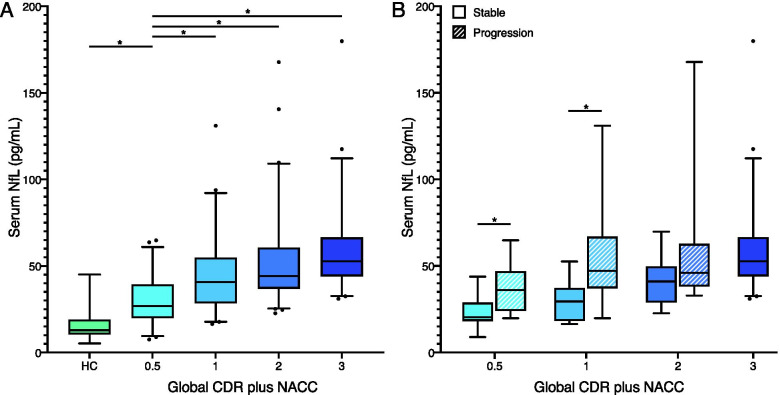
Serum NfL levels according to the global CDR plus NACC FTLD. Serum NfL levels (pg/mL) according to **A** global CDR plus NACC FTLD and **B** global CDR plus NACC FTLD divided in progressors and non-progressors. CDR plus NACC FTLD = clinical dementia rating plus National Alzheimer’s Coordinating Center behaviour and language domains; NfL = neurofilament light; HC = healthy controls. Box plots represent median and interquartile range, while whiskers represent 5–95% percentiles. *Significant difference between groups (for panel **A**, only differences with CDR plus NACC FTLD = 0.5 are shown) after Bonferroni-corrected post hoc tests

Serum NfL concentrations were significantly increased in all FTD subgroups compared with HC (age corrected ANCOVA, *p* < 0.001, *η*^2^ = 0.58). Patients with prodromal FTD had higher serum NfL levels compared with HC (average difference 14.5, 95% CI 2.9 to 26.1 pg/mL) but lower NfL levels than in patients with mild FTD (average difference − 15.5, 95% CI − 28.4 to − 2.7 pg/mL). No significant differences in serum NfL levels were observed between mild and moderate FTD or between moderate and severe FTD (see Fig. 3A).

We did not observe significant differences in serum NfL levels in the different phenotypes across global CDR plus NACC FTLD severity groups.

### Disease progression

In participants with a 1-year follow-up, in the prodromal FTD group (*n* = 84), we observed conversion to dementia in 43 patients (51.2%), whereas 41 patients (48.8%) remained in the prodromal phase. Of those converting to dementia, 22 (26.2%) converted to mild dementia, 19 (22.6%) to moderate and 2 (2.4%) to severe dementia.

In the mild FTD group (*n* = 68), 3 patients (14.7%) reverted to prodromal FTD, 23 (23.5%) patients remained stable as mild FTD, 30 (44.1%) progressed to moderate dementia and 12 (17.6%) to severe dementia.

In the moderate FTD group (*n* = 68), 1 patient (1.5%) reverted to mild dementia, 27 (39.7%) patients remained stable as moderate FTD and 40 (58.8%) progressed to severe dementia.

**Fig. 4:**
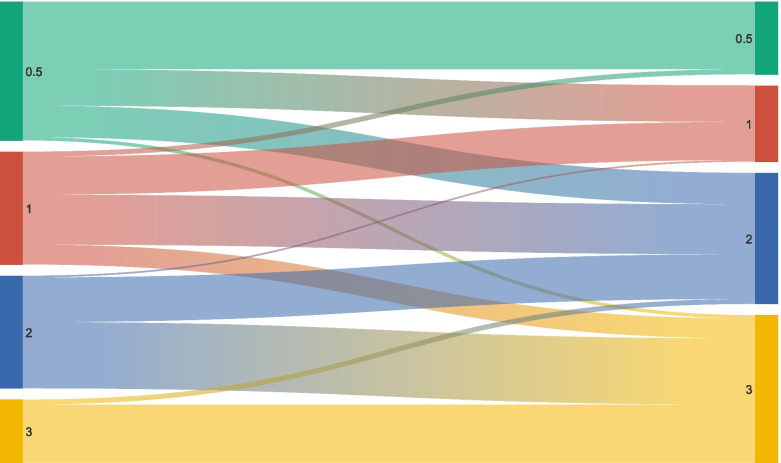
Sankey diagram showing the evolution of patients according to the global CDR plus NACC FTLD. The changes of patients over time at different time points are represented in different global CDR plus NACC FTLD groups. The height of the boxes and the thickness of the stripes are proportional to the number of patients belonging to each group and moving from each group, respectively

In the severe FTD group (*n* = 38), 3 patients (7.9%) reverted to moderate dementia whereas 35 (92.1%) remained in the same severity subgroup at follow-up (see Fig. 4). Demographic and neuropsychological characteristics of included patients for each subgroup divided in converters and non-converters are reported in Supplementary Table 2.

For each phenotype, we observed that 52.5% of prodromal bvFTD, 41.7% of prodromal avPPA and 53.8% of prodromal svPPA converted to dementia after 1-year follow-up (see Supplementary Fig. 1).

### Predictors of progression

The multilevel univariable and multivariable logistic regression models examining factors associated with progression (i.e. the transition to a higher global CDR plus NACC FTLD score at 1-year follow-up) are shown in Table 2. Serum NfL levels, MMSE and FBI scores resulted the most significant predictors in the multilevel multivariable model correctly predicting 74.0% of cases.

**Table 2:** Multilevel univariable and multivariable logistic regressions predicting likelihood of disease progression^a^

	Univariable OR (95% CI)	***P*** value	Multivariable OR (95% CI)	***P*** value
Age, years	1.00 (0.97 to 1.03)	0.927	–	–
Male sex (vs female)	0.72 (0.42 to 1.23)	0.225	–	–
Education, years	1.01 (0.94 to 1.09)	0.769	–	–
Genetic mutation (vs sporadic)	1.82 (0.86 to 3.85)	0.115	–	–
bvFTD (vs PPA)	1.30 (0.72 to 2.33)	0.387	–	–
Disease duration	1.02 (0.91 to 1.16)	0.711	–	–
Serum NfL, pg/mL	**1.07 (1.03 to 1.11)**	**< 0.001**	**1.08 (1.03 to 1.13)**	**0.003**
MMSE score	**0.93 (0.88 to 0.99)**	**0.020**	**0.79 (0.64 to 0.99)**	**0.040**
FBI score	**1.05 (1.02 to 1.08)**	**0.004**	**1.13 (1.03 to 1.24)**	**0.008**
BADL lost	1.22 (0.77 to 1.90)	0.391	–	–
IADL lost	1.16 (0.95 to 1.41)	0.149	–	–
Digit span	0.84 (0.68 to 1.03)	0.098	–	–
Trail making test part A	**1.00 (1.00 to 1.01)**	**0.029**	1.00 (1.00 to 1.01)	0.362
Trail making test part B	**1.00 (1.00 to 1.00)**	**0.015**	1.00 (1.00 to 1.01)	0.485

*bvFTD* Behavioural variant frontotemporal dementia, *PPA* Primary progressive aphasia, *NfL* Neurofilament light chain, *MMSE* Mini-Mental State Examination, *FBI* Frontal Behavioural Inventory

^a^Transition to a higher global CDR plus NACC FTLD score at 1-year follow-up

In patients classified as prodromal and mild FTD, we observed significant higher levels of serum NfL in patients who converted to mild or moderate dementia, respectively: average difference of 14.0 (95% CI 6.3 to 21.6 pg/mL), *p* = 0.001 in prodromal FTD; average difference of 26.4 (95% CI 9.1 to 43.7 pg/mL, *p* = 0.004 in mild FTD (see Fig. 3B). No significant differences in NfL levels between progressors and non-progressors were observed in the moderate FTD group.

## Discussion

The early stages of FTD are still poorly defined and likely encompass a long accrual of progressive preclinical and then prodromal changes, antedating the onset of overt dementia. The study of *at-risk* subjects in monogenic FTD has provided substantial knowledge on the earliest phases of disease, both clinically and biologically [5, 18]. Conversely, the characterization of the prodromal phases of sporadic FTD as well as the predictors of conversion to dementia are still in need of a proper definition.

The global scoring of the CDR plus NACC FTLD has been shown to be a reliable measure currently available to identify the early phases of FTD, and to have very good interrater reliability comparable to global CDR scores [8]. However, the core features of CDR plus NACC FTLD equal to 0.5, i.e. the related cognitive and behavioural traits, have only been marginally described. In this large retrospective study, we observed that in the prodromal phase, FTD patients were impaired primarily in executive functions, and presented early negative symptoms, as apathy, indifference, loss of insight, logopenia and comprehension deficits. We suggest that the trail making test part B, as already demonstrated in monogenic FTD [5], and the FBI, could be useful to identify the earliest stages of disease.

Interestingly, FTD patients were characterized by still preserved autonomy in most BADL and IADL up to moderate disease stages, suggesting that the current concept of dementia based on the impairment of activities of daily living cannot be strictly applied to symptomatic FTD.

As well as cognitive and behavioural changes, serum NfL levels have been shown to be valuable markers of disease severity in genetic and sporadic FTD [12, 14, 15, 17, 35–37]. In line with studies on genetic FTD, in this work we observed that serum NfL levels were significantly increased already in the prodromal phases of disease compared with healthy controls, but still considerably lower than patients with mild, moderate or severe FTD. Conversely, serum NfL tended to plateau in the more advance stages, with non-significant differences between mild/moderate and moderate/severe stages. These results are similar to what has been observed in the natural history of others neurodegenerative conditions, including amyotrophic lateral sclerosis, sharing common pathways with frontotemporal dementia [38, 39].

The second aim of the present study was to assess the evolution of prodromal FTD over time. We reported that, as with the notion of MCI due to Alzheimer’s disease, the concept of prodromal FTD is highly dynamic and may change over time. Indeed, in our cohort we observed that nearly 50% patients progressed from prodromal FTD to mild or moderate FTD after 1-year follow-up, with similar rates of progression in the other disease severity groups. In few cases, we also observed improvement of severity, with three patients reverting back from mild FTD to prodromal disease. Patients with prodromal avPPA showed slightly reduced conversion rates compared to prodromal bvFTD and svPPA patients. Rate of progression to dementia was higher than in MCI due to Alzheimer’s disease [40], but further studies in larger samples of prodromal FTD patients are needed.

Finally, we aimed to identify also which factors were associated with the risk of progression. We observed that serum NfL levels were the most significant predictors of conversion to dementia and disease progression, being significantly increased in patients that transitioned to a higher global CDR plus NACC FTLD score at the 1-year follow-up, both in the prodromal and mild FTD stages. NfL levels have already been shown to predict clinical decline and survival in sporadic FTD patients [12, 41, 42], but studies on the impact of NfL levels on the conversion from prodromal to dementia phases were still lacking. Similarly to what we observed in this study, NfL levels have been shown to identify mutation carriers approaching symptom onset in genetic FTD [18, 43].

These findings have important clinical implications. Knowledge of the clinical and biological characteristics of patients in the earliest disease stages is particularly relevant for counselling patients and caregivers, and for the evaluation of outcomes in FTD therapeutic trial designs. Moreover, the assessment of blood NfL levels may provide crucial advice in identifying patients at risk of progression to dementia and may be considered a reliable prognostic marker to be used in clinical trials.

## Limitations

We acknowledge that this study has some limitations. Our study tried to evaluate several clinical and behavioural aspects of prodromal FTD, but further cognitive, behavioural and motor features should be comprehensively explored in prodromal FTD. For sporadic cases, diagnosis was based on clinical features and laboratory/imaging evaluations and might thus be occasionally incorrect. Serum NfL measurements and extensive 1-year follow-up evaluations were not available for all patients; nevertheless, all patients classified as prodromal FTD had a follow-up evaluation that confirmed eventual conversion to dementia or were carriers of a pathogenic FTD mutation. Moreover, the longitudinal evaluation was limited to 1 year, partially restricting further considerations on disease evolution. The unique strength of our study was the extensive and longitudinal characterization of a large number of sporadic prodromal FTD patients.

## Conclusions

In conclusion, this study tried to clarify the initial, prodromal phases of FTD, analysing disease progression rates and markers of conversion to dementia, having crucial implications for counselling patients and caregivers, and providing a proper framework for the future design of disease-modifying treatment trials in sporadic FTD.

## Supplementary Information

**Additional file 1: Supplementary Table 1.** Demographic and clinical characteristics of FTLD patients grouped according to the global CDR plus NACC FTLD and mutational status. **Supplementary Table 2.** Demographic and clinical characteristics of FTLD patients grouped according to the global CDR plus NACC FTLD and conversion at 12-months follow-up. **Supplementary Figure 1.** Sankey diagram showing the evolution of patients according to the global CDR plus NACC FTLD and phenotype.

## Data Availability

All study data, including raw and analysed data, and materials will be available from the corresponding author, B.B., upon reasonable request.
