# Supplementary material for: Prodromal frontotemporal dementia: clinical features and predictors of progression
Source: Alzheimers Res Ther. 2021 Nov 15;13:188. doi: 10.1186/s13195-021-00932-2 (PMC8594126; doi:10.1186/s13195-021-00932-2)
Supplement: Supplementary file 1 — Additional file 1: Supplementary Table 1. Demographic and clinical characteristics of FTLD patients grouped according to the global CDR plus NACC FTLD and mutational status. Supplementary Table 2. Demographic and clinical characteristics of FTLD patients grouped according to the global CDR plus NACC FTLD and conversion at 12-months follow-up. Supplementary Figure 1. Sankey diagram showing the evolution of patients according to the global CDR plus NACC FTLD and phenotype. [file 13195_2021_932_MOESM1_ESM.pdf]

Supplementary Table 1. Demographic and clinical characteristics of FTLD patients grouped according to the global CDR plus NACC FTLD and mutational status.

	Global CDR plus NACC FTLD			
**Variable**	**0.5 sporadic**	**0.5 genetic**	**1 sporadic**	**1 genetic**
Number	112	26	108	22
Age, years	66.8 (59.8-71.9)	61.4 (55.9-66.7)†	69.6 (63.6-74.8)	62.8 (56.6-70.7)†
Sex male, n (%)	66 (58.9)	14 (53.8)	56 (51.9)	16 (72.7)
Education, years	8.0 (5.0-13.0)	9.0 (7.8-13.0)	8.0 (5.0-13.0)	8.0 (5.0-13.0)
Age at onset, years	65.0 (57.3-69.0)	57.5 (53.8-64.0)†	66.5 (61.0-72.0)	60.5 (54.0-68.3)†
Disease duration, years	1.9 (1.3-3.1)	1.7 (1.1-3.0)	2.1 (1.3-3.3)	2.0 (1.5-2.8)
Serum NfL (pg/mL)*	27.2 (20.1-37.7)	24.8 (19.1-46.5)	39.0 (27.5-48.7)	66.9 (52.4-78.0)†
bvFTD/avPPA/svPPA, n	75/18/19	17/7/2	75/20/13	14/7/1
MMSE	25.9 (24.4-26.9)	26.0 (24.0-27.9)	23.2 (20.4-26.0)	22.8 (20.2-25.5)
FBI	7.0 (3.0-10.8)	9.0 (4.0-12.5)	11.5 (6.3-17.0)	12.0 (7.8-21.3)
BADL lost	0.0 (0.0-0.0)	0.0 (0.0-0.0)	0.0 (0.0-0.0)	0.0 (0.0-0.0)
IADL lost	0.0 (0.0-0.0)	0.0 (0.0-0.0)	0.0 (0.0-1.0)	0.0 (0.0-1.0)
Short story	9.5 (8.0-13.0)	9.0 (6.8-11.6)	8.5 (5.8-10.9)	8.1 (6.6-11.1)
Rey figure copy	30.5 (26.4-32.8)	29.7 (23.5-32.8)	27.9 (22.4-32.7)	26.4 (18.1-32.3)
Rey figure recall	11.3 (8.5-16.5)	13.8 (10.5-17.8)	9.8 (6.5-13.3)	9.6 (6.5-13.4)
Phonological fluency	24.0 (19.0-29.8)	22.6 (14.2-28.3)	20.5 (15.0-29.0)	16.0 (9.8-26.8)
Semantic fluency	31.5 (24.3-36.8)	31.0 (24.0-38.3)	27.0 (20.0-33.0)	26.6 (18.5-32.5)
Digit span	5.0 (4.5-5.7)	5.0 (4.6-5.8)	4.9 (4.3-5.5)	4.5 (3.5-5.9)
Token test	30.1 (28.0-32.3)	28.7 (27.4-31.3)	28.3 (26.4-30.1)	28.3 (22.8-30.5)
Trail making test part A	51.0 (28.0-73.0)	48.5 (27.0-72.5)	64.0 (42.3-108.0)	59.0 (39.0-115.8)
Trail making test part B	187.5 (105.3-345.5)	238.0 (92.0-401.3)	324.0 (121.3-408.8)	394.0 (176.5-442.5)
Clock drawing	8.0 (6.0-9.0)	8.0 (6.0-9.0)	7.0 (5.0-8.8)	8.0 (5.0-10.0)
**Variable**	**2 sporadic**	**2 genetic**	**3 sporadic**	**3 genetic**
Number	141	34	94	26
Age, years	66.7 (61.0-71.9)	61.0 (54.7-66.5)†	67.7 (61.1-74.6)	65.5 (61.5-69.9)
Sex male, n (%)	76 (53.9)	15 (44.1)	43 (45.7)	10 (38.5)
Education, years	8.0 (5.0-8.0)	10.0 (7.3-13.0)	8.0 (5.0-11.3)	8.0 (5.0-13.0)
Age at onset, years	63.0 (58.0-69.0)	58.0 (51.8-64.0)†	64.0 (56.8-71.0)	61.5 (59.5-67.0)
Disease duration, years	2.6 (1.7-4.2)	2.4 (1.6-3.2)	3.2 (2.0-5.2)	2.6 (1.8-4.2)
Serum NfL (pg/mL)*	43.4 (36.1-59.8)	55.3 (37.8-108.8)	47.7 (39.9-55.4)	74.1 (60.1-88.8)†
bvFTD/avPPA/svPPA, n	99/25/17	22/10/2	73/13/8	17/8/1
MMSE	20.0 (15.0-23.0)	20.6 (16.7-24.0)	11.6 (4.0-19.1)	7.5 (0.0-14.5)
FBI	19.0 (11.5-27.0)	22.5 (14.8-25.3)	30.5 (24.0-37.0)	32.5 (25.3-36.3)
BADL lost	0.0 (0.0-1.0)	0.0 (0.0-1.0)	3.0 (1.0-4.0)	3.0 (2.0-5.0)
IADL lost	2.0 (1.0-3.0)	2.0 (0.0-4.0)	5.0 (3.8-7.0)	6.0 (5.0-7.3)
Short story	6.0 (3.5-8.0)	6.2 (3.5-8.4)	5.2 (3.8-7.5)	5.7 (4.4-8.1)
Rey figure copy	20.8 (13.6-29.0)	22.0 (17.6-28.3)	13.6 (6.9-20.1)	10.0 (6.8-13.6)
Rey figure recall	6.1 (3.2-9.7)	7.1 (4.4-9.6)	5.2 (3.1-7.3)	5.8 (4.0-7.5)
Phonological fluency	15.0 (9.6-21.5)	12.0 (7.0-20.5)	10.1 (5.0-15.6)	7.4 (2.3-11.5)
Semantic fluency	18.0 (13.0-25.0)	22.3 (14.5-26.8)	10.9 (5.3-18.1)	7.4 (3.1-17.0)
Digit span	4.5 (3.8-5.5)	4.5 (4.0-5.0)	3.8 (2.8-4.5)	3.4 (2.8-4.0)
Token test	24.1 (19.3-28.0)	23.7 (21.3-27.5)	18.8 (14.7-24.6)	17.0 (11.0-21.6)
Trail making test part A	115.0 (68.0-297.0)	86.5 (48.5-173.3)	212.5 (131.0-394.5)	321.5 (184.5-387.0)
Trail making test part B	403.0 (345.0-426.5)	407.5 (299.0-447.8)	398.5 (364.8-422.3)	421.5 (395.5-463.3)
Clock drawing	5.0 (3.0-6.0)	5.0 (3.0-7.0)	3.0 (1.0-4.3)	2.0 (0.0-3.3)

NfL = neurofilament light chain; CDR plus NACC FTLD SB = clinical dementia rating plus National Alzheimer's Coordinating Center behaviour and language domains FTLD sum-of-boxes; MMSE = mini mental state examination; FBI = frontal behavioural inventory; BADL = basic activities of daily living; IADL = instrumental activities of daily living. Results are expressed as median (interquartile range), unless otherwise specified.

*results for serum NfL are reported for a subgroup of patients; †significant difference vs sporadic cases at Mann-Whitney U test or Fisher's test, as appropriate, FDR-corrected for multiple comparisons.

Supplementary Table 2. Demographic and clinical characteristics of FTLD patients grouped according to the global CDR plus NACC FTLD and conversion at 12-months follow-up.

	Global CDR plus NACC FTLD						
**Variable**	**0.5 non-converters**	**0.5 converters**	**1 non-converters**	**1 converters**	**2 non-converters**	**2 converters**	**3**
Number	41	43	26	42	28	40	38
Age, years	66.7 (60.4-72.1)	65.1 (56.9-70.7)	68.7 (63.9-75.4)	64.9 (57.5-74.1)	62.6 (57.7-68.8)	66.6 (61.0-71.9)	65.8 (62.0-73.4)
Sex male, n (%)	22 (53.7)	27 (62.8)	17 (65.4)	19 (45.2)	16 (57.1)	16 (40.0)	18 (47.4)
Education, years	8.0 (5.0-13.0)	8.0 (5.0-13.0)	8.0 (5.0-8.3)	8.0 (5.0-13.0)	7.0 (5.0-8.0)	8.0 (5.0-8.0)	5.0 (5.0-11.0)
Age at onset, years	64.0 (57.5-69.5)	64.0 (56.0-69.0)	65.0 (61.8-69.8)	63.0 (55.8-71.3)	60.0 (55.3-65.8)	62.5 (58.0-68.0)	63.5 (56.0-68.8)
Disease duration, years	2.2 (1.3-3.5)	1.9 (1.2-2.8)	2.1 (1.2-3.5)	2.2 (1.4-3.2)	1.8 (1.4-2.6)	2.8 (1.9-4.4)	2.4 (1.6-4.4)
Monogenic disease, n (%)	6 (14.6)	11 (25.6)	4 (15.4)	7 (16.7)	2 (7.1)	8 (20.0)	9 (23.7)
Serum NfL (pg/mL)*	20.4 (18.1-28.9)	36.0 (24.0-47.0)†	29.5 (18.2-37.3)	47.2 (37.0-66.9)†	41.1 (28.9-49.8)	46.0 (38.1-62.8)	51.9 (44.1-76.9)
bvFTD/avPPA/svPPA, n	28/7/6	31/5/7	20/4/2	27/8/7	21/5/2	26/8/6	34/3/1
MMSE	26.2 (25.3-27.5)	25.9 (23.9-26.9)	25.0 (23.2-26.1)	22.5 (19.8-25.5)	20.0 (16.9-22.7)	20.0 (14.8-22.3)	12.9 (2.7-16.0)
FBI	5.0 (2.0-9.0)	10.0 (7.0-13.0)†	11.5 (5.0-19.0)	13.0 (8.0-17.5)	16.0 (12.0-22.8)	19.5 (10.0-26.8)	30.5 (23.8-36.0)
BADL lost	0.0 (0.0-0.0)	0.0 (0.0-0.0)	0.0 (0.0-0.0)	0.0 (0.0-0.0)	0.0 (0.0-0.8)	0.0 (0.0-0.0)	3.0 (1.0-5.0)
IADL lost	0.0 (0.0-0.0)	0.0 (0.0-0.0)	0.0 (0.0-0.0)	0.0 (0.0-1.0)	2.0 (1.3-3.8)	2.0 (1.0-4.0)	5.0 (4.0-7.0)
Short story	12.5 (8.5-15.0)	9.1 (6.5-11.5)†	9.8 (6.3-14.6)	7.0 (4.0-10.5)	5.6 (4.1-8.0)	5.2 (0.6-7.5)	5.7 (2.8-8.1)
Rey figure copy	30.5 (27.7-32.7)	27.5 (23.3-31.8)	29.0 (24.7-32.4)	27.7 (21.2-33.6)	20.9 (17.4-29.4)	19.2 (12.9-26.7)	10.7 (6.2-19.2)
Rey figure recall	16.3 (10.7-19.5)	10.5 (7.3-15.0)†	9.8 (7.1-14.8)	9.2 (3.7-13.6)	6.7 (1.3-10.0)	5.4 (0.4-8.5)	4.5 (2.7-7.4)
Phonological fluency	28.0 (22.0-36.5)	23.0 (15.0-28.0)†	25.5 (16.8-31.5)	17.0 (11.0-28.1)	17.5 (9.9-23.0)	11.0 (8.3-20.5)	11.5 (4.1-19.2)
Semantic fluency	35.0 (27.5-42.0)	29.0 (26.0-36.0)†	31.0 (20.8-38.3)	26.0 (18.5-32.0)	22.5 (15.3-28.5)	15.0 (11.4-22.8)	11.3 (3.1-19.8)
Digit span	5.0 (4.4-6.0)	5.2 (4.5-5.8)	5.3 (4.5-5.6)	4.5 (3.8-5.8)	5.0 (4.0-5.5)	4.3 (3.4-5.5)	3.5 (2.4-4.5)
Token test	31.0 (29.4-33.0)	29.4 (27.8-32.5)	29.3 (27.5-31.6)	27.0 (23.1-29.6)†	24.4 (22.4-28.9)	23.8 (19.4-27.0)	18.1 (12.0-22.9)
Trail making test part A	49.0 (24.5-73.5)	51.0 (28.0-80.0)	53.0 (26.5-73.5)	68.5 (41.8-111.0)	101.0 (50.5-212.0)	141.5 (70.0-472.0)	353.5 (168.0-476.0)
Trail making test part B	144.0 (73.5-253.0)	223.0 (140.0-396.0)†	306.5 (94.8-409.0)	349.5 (157.0-432.3)	408.0 (315.3-430.5)	411.0 (393.8-431.5)	405.0 (370.0-424.0)
Clock drawing	9.0 (7.0-10.0)	7.0 (5.0-9.0)†	7.0 (5.0-9.0)	7.0 (4.0-8.0)	5.0 (4.0-6.0)	4.5 (2.0-6.0)	3.0 (1.0-5.0)

NfL = neurofilament light chain; CDR plus NACC FTLD SB = clinical dementia rating plus National Alzheimer's Coordinating Center behaviour and language domains FTLD sum-of-boxes; MMSE = mini mental state examination; FBI = frontal behavioural inventory; BADL = basic activities of daily living; IADL = instrumental activities of daily living. Results are expressed as median (interquartile range), unless otherwise specified.

*results for serum NfL are reported for a subgroup of patients; †significant difference vs non-converters at Mann-Whitney U test or Fisher's test, as appropriate, FDR-corrected for multiple comparisons.

Supplementary Figure 1. Sankey diagram showing the evolution of patients according to the global CDR plus NACC FTLD and phenotype.

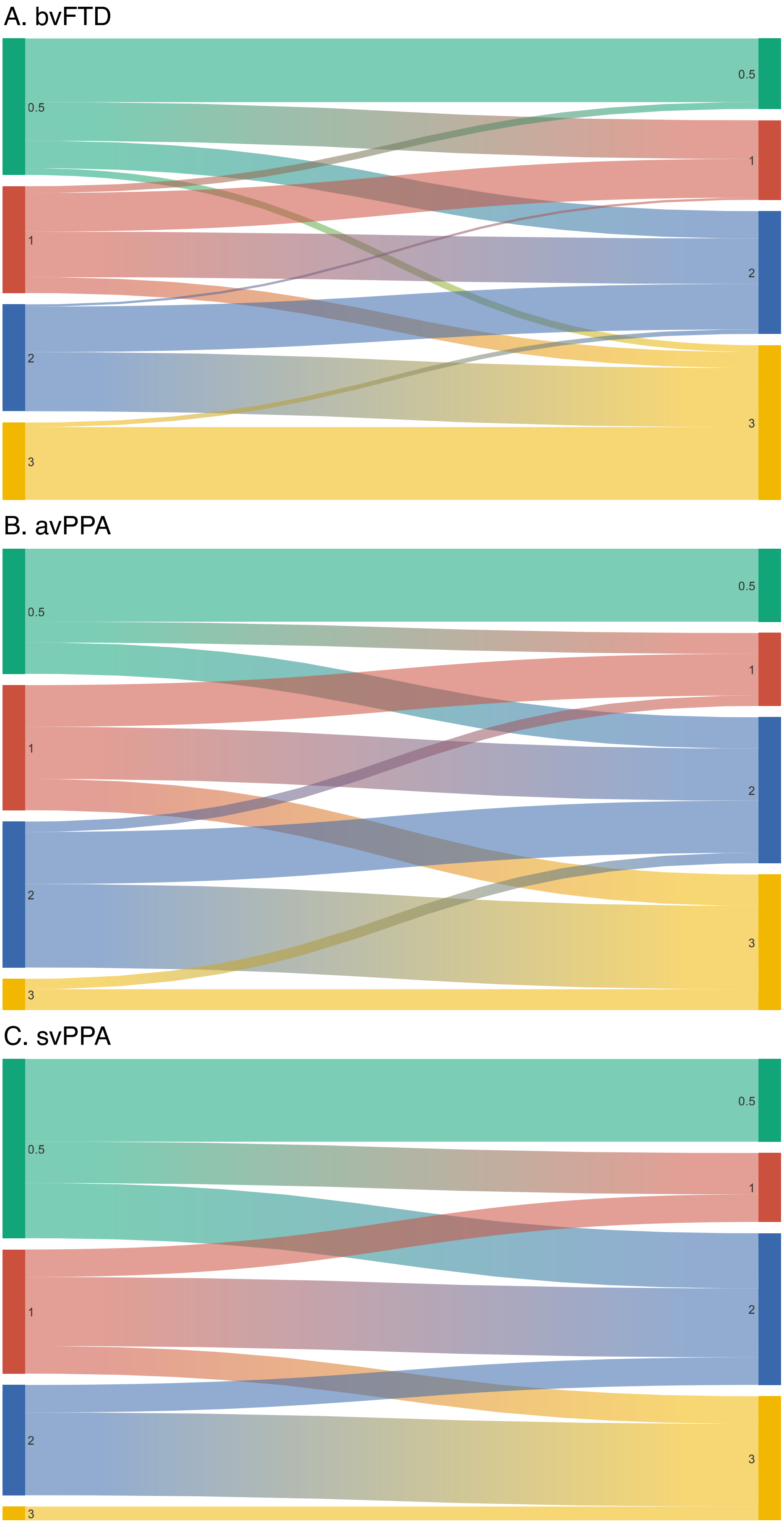

The changes of patients over time at different time points is represented in different global CDR plus NACC FTLD groups. The height of the boxes and the thickness of the stripes are proportional to the number of patients belonging to each group and moving from each group, respectively. bvFTD = behavioural variant frontotemporal dementia; avPPA = agrammatic variant primary progressive aphasia; svPPA = semantic variant primary progressive aphasia.
